# Surface Characterization and Photoluminescence Properties of Ce^3+^,Eu Co-Doped SrF_2_ Nanophosphor

**DOI:** 10.3390/ma8052361

**Published:** 2015-04-30

**Authors:** Mubarak Y. A. Yagoub, Hendrik C. Swart, Luyanda L. Noto, Peber Bergman, Elizabeth Coetsee

**Affiliations:** 1 Department of Physics, University of the Free State, PO Box 339, Bloemfontein, ZA 9300, South Africa; E-Mails: yagoubm@ufs.ac.za (M.Y.); luyanda.noto@gmail.com (L.N.); 2 Department of Physics, Sudan University of Science and Technology, Khartoum 11113, Sudan; 3 Department of Physics, Chemistry and Biology, Linköping University, Linköping S-581 83, Sweden; E-Mail: peber@ifm.liu.se

**Keywords:** SrF_2_, cerium, TOF-SIMS, XPS, shake-down, energy transfer

## Abstract

SrF_2_:Eu,Ce^3+^ nanophosphors were successfully synthesized by the hydrothermal method during down-shifting investigations for solar cell applications. The phosphors were characterized by X-ray diffraction (XRD), scanning Auger nanoprobe, time of flight-secondary ion mass spectrometry (TOF-SIMS), X-ray photoelectron spectroscopy (XPS) and photoluminescence (PL) spectroscopy. XRD showed that the crystallite size calculated with Scherrer’s equation was in the nanometre scale. XPS confirmed the formation of the matrix and the presence of the dopants in the SrF_2_ host. The PL of the nanophosphor samples were studied using different excitation sources. The phenomenon of energy transfer from Ce^3+^ to Eu^2+^ has been demonstrated.

## 1. Introduction

Strontium fluoride (SrF_2_) is one of the most widely used optical materials because of its interesting luminescent, optical, and physical properties. It has a wide band gap, low phonon energy, low refraction index, high radiation resistance, and good mechanical strength [1,2]. The photoluminescence properties of SrF_2_ doped by Ln^3+^ ions have been extensively investigated in which charge compensation is required when Ln^3+^ ions substitute Sr^2+^ cation. This gives rise to a rich multisite structure. It has therefore been considered as a good phosphor host material that can be doped by a number of lanthanide ions for various luminescent applications [1,2,3,4]. SrF_2_ host material doped with Ce^3+^ lanthanide ions is an example of a phosphor material that is extensively being investigated specifically for light amplification [5,6]. Some of these light amplification studies proposed that the SrF_2_:Ce^3+^ phosphor material could be a promising scintillator [5]. Shendrik *et al.* [5] reported efficient scintillation light output of SrF_2_:Ce^3+^ with high temperature stability suggesting that this material can be applied in well-logging scintillation detectors. They have also reported that the optimal Ce^3^^+^ doping level for maximum luminescence was 0.3 mol% if prepared by the Stockbarger method. Ce^3+^ ions in SrF_2_ showed a fully allowed broad band 4f−5d transition [5] and this transition strongly absorbs UV radiation that results in a high absorption coefficient.

In the other hand, several previous studies have described the luminescence of Eu^3+^ doped materials as a good downshifting ion [7,8,9,10]. Gao *et al.* [7] reported luminescence due to transitions from the ^5^D_0_ excited level to the ^7^F_J_ levels, where spectral conversion of 325–550 nm light to 570–710 nm light has been demonstrated. In our previous investigation of SrF_2_:Eu we reported the emissions from both the Eu oxidation states (Eu^3+^ and Eu^2+^) where emission from 400 to 710 was observed [10]. X-ray photoelectron spectroscopy (XPS) results confirmed that the samples contained both Eu^2+^ and Eu^3+^ ions. The Eu^3+^ ion doped materials emits narrow emission peaks in the range of the orange-red emission with large Stokes shifts (>150 nm) that originates from the 4f−4f weak absorption transitions [11,12], whereas the 4f−5d absorption transition of the Eu^3+^ ion in SrF_2_ is situated at the far ultraviolet region, which can be less accessible. In some applications, high or suitable absorption cross-section is needed and this requires a sensitizer with a high absorption coefficient [2,9,13]. Therefore, the presence of the Eu^2+^ and Eu^3+^ ions in the SrF_2_ host greatly enhanced the emission intensity of Eu^3+^ at high concentrations [10]. In this work, Ce^3+^ singly and co-doped Eu in SrF_2_ was prepared by using the hydrothermal method. The surface and photoluminescence properties are discussed. 

## 2. Results and Discussion

### 2.1. Structure Analysis

#### 2.1.1. X-Ray Diffraction (XRD)

**Figure 1:**
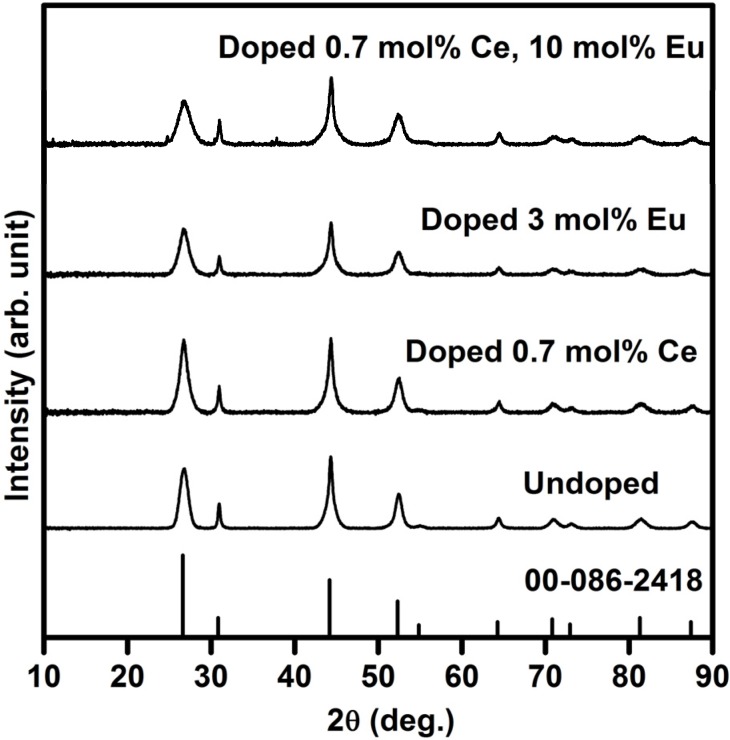
XRD patterns of pure and doped SrF_2_.

Figure 1 shows the XRD patterns of un-doped and doped SrF_2_ as well as the standard data for SrF_2_ from card 00-086-2418. Doping with Ce- or Eu ions as well as the co-doped systems result in a small shift to higher angles with comparison to the un-doped sample and the standard data. This can be attributed to the radius difference between Eu (Eu^2+^ is 0.125 nm, Eu^3+^ is 0.107 nm), Ce^3+^ (0.114 nm) and Sr^2+^ (0.126 nm) ions, which confirms that Eu- and Ce ions are successfully incorporated into the SrF_2_ lattice. It should be mentioned that doping with Eu- and Ce ions (up to 10 mol%) does not change the structure of the SrF_2_ host in this study. The calculated SrF_2_ lattice parameter is found to be (5.785 ± 0.005) Å and this agreed well with the reported value of (5.7996 ± 0.0001) Å [14]. 

The estimated average crystallite size (S) for pure and doped SrF_2_ is calculated by using the diffraction peaks and Scherrer’s equation [15], S = 0.9λ/βcosθ. S is the average crystallite size of the SrF_2_ particles, λ is the wavelength of the X-rays (0.154 nm) and β is the full-width at half maximum of the X-ray peak at the Bragg angle θ. The average crystallite size of the pure SrF_2_ was found to be 7.6 nm. The XRD peaks broaden with increasing the dopants ions (see Figure 1). The broadening of the XRD peaks were also observed by other groups [16,17]. H.A.A. Seed Ahmed *et al.* [16] attributed the XRD peak broadening to impurity broadening. Whereas, F. Wang *et al.* [17] assigned the XRD peak broadening to reduction in the nanoparticle size of the matrix. In our previous investigation of Eu doped SrF_2_ samples, we assigned the XRD broadening as a result of a decrease in particle size of the matrix, which agreed well with F. Wang *et al.* [10]. Therefore, in the current study we can also assign these peaks’ broadening to reduction in particle size of the matrix. The particle size reduced up to 3.9 nm for the SrF_2_ sample that was doped with 0.7 mol% Ce^3+^ and 10 mol% Eu.

#### 2.1.2. Auger and TOF SIMS analysis

**Figure 2:**
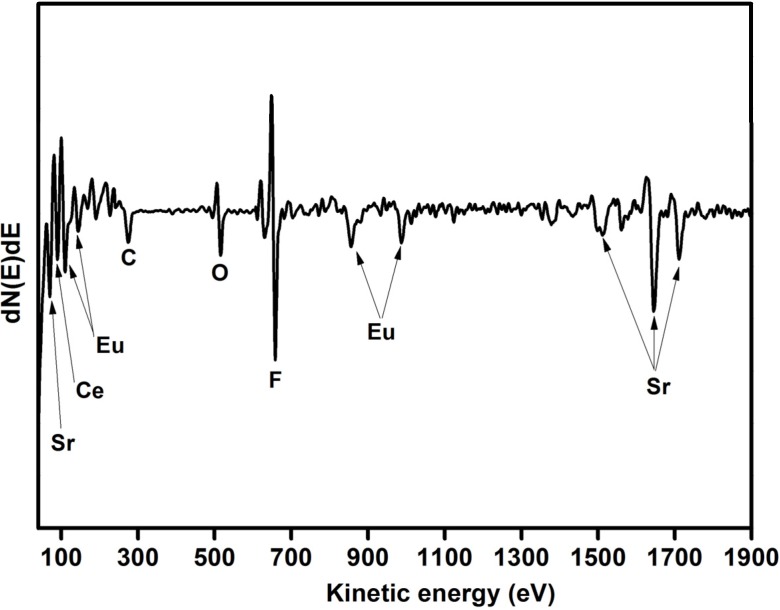
Auger spectrum of Ce and Eu co-doped SrF_2_.

An Auger profile of Ce and Eu co-doped SrF_2_ was done to identify the sample’s composition. The Auger spectrum of the SrF_2_:Ce^3+^,Eu is presented in Figure 2. The Auger peaks at 71, 1515, 1644 and 1713 eV are assigned to Sr while the F peak is situated at 656 eV [18]. The Auger spectrum not only confirmed the formation of the host matrix, but also showed the presence of the dopants. The Eu peaks were at 111, 142, 853 and 985 eV, while the peak at 89 eV corresponds to Ce. In addition C and O were also observed. The C contamination is attributed to adventitious hydrocarbons and the O is considered to be a common impurity in a fluoride compound [19,20]. The presence of the O in the sample did not change the structure of the sample (see Figure 1). Therefore, the O contamination was due to adventitious impurity species in the surface rather than oxygen impurity in the SrF_2_ matrix.

It could clearly be seen, not shown, that both the Ce and Eu ions were distributed quite homogeneously over the entire surface area of the Ce and Eu co-doped SrF_2_. That indicated that the dopants were uniformly distributed in the SrF_2_ matrix during the hydrothermal synthesis method.

#### 2.1.3. X-ray Photoelectron Spectroscopy (XPS)

**Figure 3:**
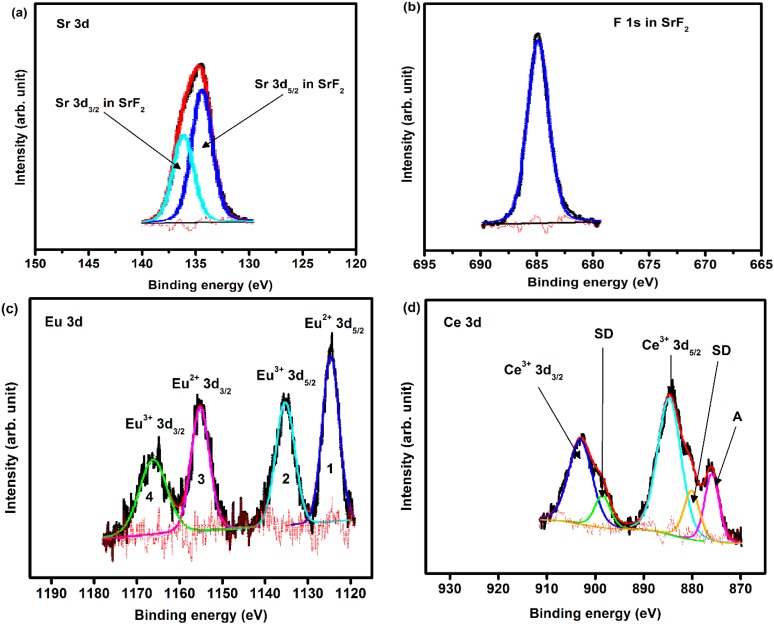
High resolution XPS peaks of (**a**) Sr 3d; (**b**) F 1s; (**c**) Eu 3d; and (**d**) Ce 3d for SrF_2_:Ce,Eu phosphors powder.

XPS measurements have been done in order to investigate the chemical composition and bonding state of the SrF_2_:Ce,Eu phosphor powders. A higher dopant concentration (5 mol% for both Eu and Ce) was used in order to obtain a reasonable signal from the dopants. Figure 3 shows the peak fits for the (a) Sr 3*d*, (b) F 1*s*, (c) Eu 3*d* and (d) Ce 3*d* high resolution XPS peaks. The results also confirmed the presence of the host matrix elements (Sr and F) as well as the dopants (Eu and Ce) to their corresponding binding energies. During the peaks fit procedure, the C 1*s* peak at 284.8 eV was taken as a reference for all charge shift corrections. This is done because the C 1*s* peak resulted from hydrocarbon contamination and its binding energy generally remains constant, irrespective to the chemical state of the sample. In addition to that, all the Gaussian percentages were assumed to have a combined Gaussian-Lorentzian shape. The high resolution XPS peak for the Sr 3*d* showed two individual peaks. These two peaks are assigned to Sr 3*d* in SrF_2_ that originate from the spin-orbit splitting 3*d*_5/2_ (133.5 eV) and 3*d*_3/2_ (135.3 eV), while the F 1*s* peak is situated at 684.7 eV. The spin-orbit splitting of Sr 3*d* is about 1.78 eV, it is in a good agreement with reported value of 1.75 eV [21].

The peak deconvolution for the Eu 3*d* high resolution XPS peaks are shown in Figure 3c. The 3*d* level of Eu ion is composed of four peaks. These four peaks can be attributed to Eu^3+^ and Eu^2+^ spin-orbit splitting 3*d*_5/2_ and 3*d*_3/2_ core level, respectively [21,22,23,24]. The spin-orbit splitting for both oxidation states Eu^3+^ and Eu^2+^ is about 29.96 eV. The Eu 3*d* results showed good agreement with our previous XPS investigation of SrF_2_:Eu phosphors powder where Eu composed of its two oxidation states (Eu^2+^ and Eu^3+^) [10]. 

The Ce 3*d* high resolution peak is shown in Figure 3d. The strong peaks correspond to the photoemission from the Ce^3+^ 3*d* state. Due to the spin-orbit interaction, the Ce^3+^ 3*d* photoemission peak consisted of two peaks that are assigned to the 3*d*_3*/*2_ and 3*d*_5*/*2_ peaks with 4f^1^ final states, with an intensity ratio I(3*d*_5*/*2_)*/*I(3*d*_3*/*2_) = 3*/*2 [22,25,26]. The spin-orbit splitting value (*≈*18.15 eV) is in good agreement with the estimated value (*≈*18.10 eV). The energy peaks labelled SD are due to the strong Coulomb interaction between photoemission in the 3*d* level and electrons located near the Fermi level. These peaks originate from the screening of the 3*d* level by valence band electrons to the 4*f* states [22]. This is possible due to hybridization of the Ce 4*f* level with the conduction band states [26]. In the photoemission nomenclature, these peaks are a result from what is called, shake-down process [22]. The 3*d* shake-down peaks behave the same as the 3d spin-orbit splitting peaks but they are a result from the 3d^9^f^2^ final state. Therefore, the SD peaks can be assigned to the 3*d*_3*/*2_ and 3*d*_5*/*2_ XPS peaks with 4f^2^ final states and this is in accordance with previous work done in Ce [25,26]. The shoulder peaks marked as A is related to the F KLL Auger electron peak. The XPS peak positions, area distributions and chemical bonding for all the peaks in as-prepared SrF_2_:Ce,Eu are tabulated in Table 1.

**Table 1:** XPS peak position, area distribution and chemical bonding of as-prepared SrF_2_:Ce,Eu phosphor powder.

Element	B.E (±0.1 eV)	Area distribution	Interpretation
F1s	684.7	2688	F in SrF_2_
Sr3d	133.5	1986	Sr 3d_5/2_ in SrF_2_
	135.3	1311	Sr 3d_3/2_ in SrF_2_
Eu3d	1123.3	1613	Eu^2+^ 3d_5/2_ in fluoride
	1133.05	1372	Eu^3+^ 3d_5/2_ in fluoride
	1153.2	1064	Eu^2+^ 3d_3/2_ in fluoride
Ce3d	1163.0	905	Eu^3+^ 3d_3/2_ in fluoride
	880.3	1296	Shake-down satellite
	884.8	5141	Ce^3+^ 3d_5/2_ in fluoride
	898.5	855	Shake-down satellite
	903.0	3393	Ce^3+^ 3d_3/2_ in fluoride
	876.1	1592	F KL_1_L_1_ Auger electron peak

### 2.2. Photoluminescence Spectroscopy

#### 2.2.1. SrF_2_:Ce^3+^

**Figure 4:**
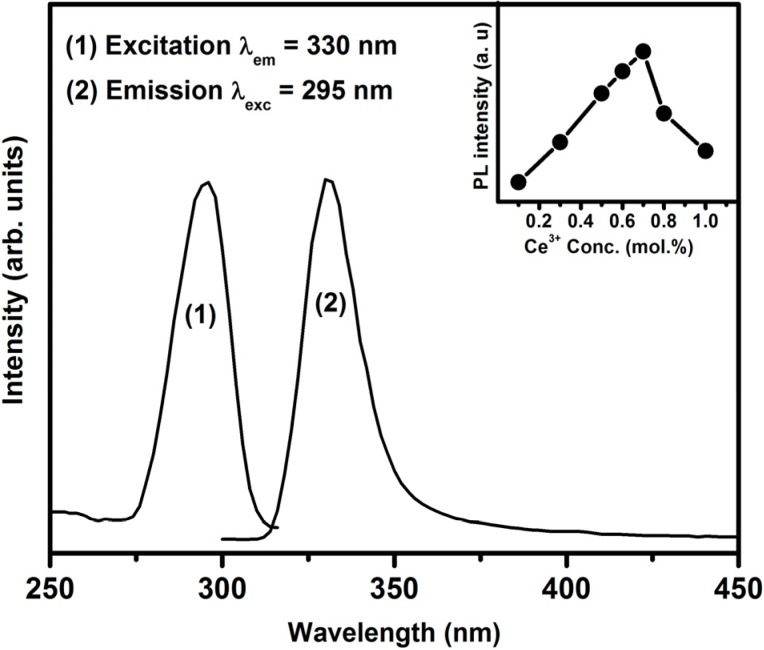
Excitation and emission spectra of the SrF_2_:Ce^3+^ (0.7 mol%) nanophosphor. The inset shows the 5d–4f transition’s emission intensity as a function of Ce^3+^ concentration.

The emission and excitation spectra of the Ce^3+^ singly doped SrF_2_ nanophosphor are shown in Figure 4. The excitation spectrum consists of a prominent peak that is centred at 295 nm. This peak has been previously assigned to Ce^3+^:4f–5d excitation transition in SrF_2_ [27]. By exciting the samples by 295 nm, a broad band emission peak is observed, which is attributed to the inter-configuration 5d^1^−4f^1^ allowed transition of Ce^3+^ ions. The inset graph in Figure 4 shows the emission intensity variation as a function of the Ce^3+^ concentration. The maximum luminescence intensity occurred for the sample doped with 0.7 mol% and a further increase in concentration resulted in a decrease in Ce^3+^ emission intensity. A previous study done by R. Shendrik *et al.* [5] on the SrF_2_:Ce^3+^ sample reported that Ce^3+^ has a broad emission band that consist of two emission peaks (Ce^3+^ 5d to 4f ground state (^2^F_7/2_ and ^2^F_5/2_)) and the maximum intensity was observed at a Ce^3+^ dopant concentration of 0.3 mol%. In this study, the peaks were broadened and they fully overlapped, which might be the reason that only one broad peak was observed.

#### 2.2.2. SrF_2_:Ce,Eu

**Figure 5:**
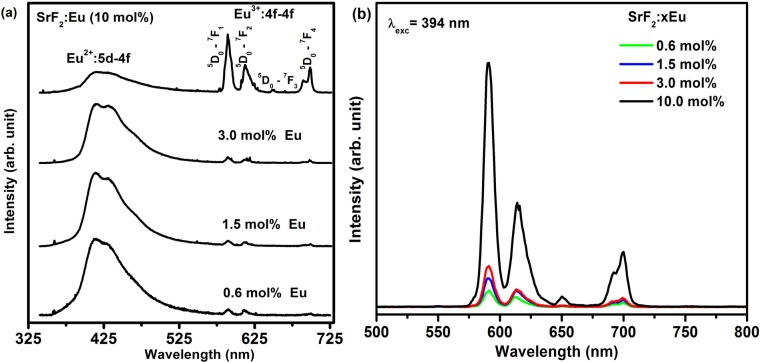
Photoluminescence spectra of SrF_2_:xEu excited by (**a**) using the He-Cd laser system with 325 nm excitation wavelength and (**b**) the Cary Eclipse with a wavelength of 394 nm.

Figure 5a shows the PL emission spectra of SrF_2_:Eu obtained by using the He-Cd laser PL system with a 325 nm excitation wavelength. The spectra clearly consist of a broad emission band that is centred at 416 nm with narrow bands in the range of 550–710 nm. The broad emission band is assigned to the inter-configuration 4f^6^5d^1^–4f^7^ allowed transition of Eu^2+^ [11,12] and the narrow emission bands to the Eu^3+^ emission originating from the 4f–4f transition [28]. The Eu^3+^ emission consists of orange–red emission bands that is attributed to the ^5^D_0_→^7^F_J_ transitions (*J* = 1, 2, 3, 4). This implies that the SrF_2_:Eu samples consist of both Eu oxidation states (Eu^2+^ and Eu^3^^+^), with their emission ranging from 400 to 710 nm [10]. The Eu^3+^ emission bands increased with an increase in the Eu dopant concentration in the SrF_2_ matrix. This can also be seen in Figure 5b, where the emission of Eu^3+^ excited by 394 nm is portrayed. The PL emission intensity increased slightly at the lower concentrations but then increased dramatically at 10 mol%. The presence of both Eu oxidation states therefore strongly enhanced the emission intensity of the Eu^3+^ ions. Detailed investigations on the luminescence phenomenon of Eu^3+^ and Eu^2+^ have previously been studied by various workers [10,29,30,31].

**Figure 6:**
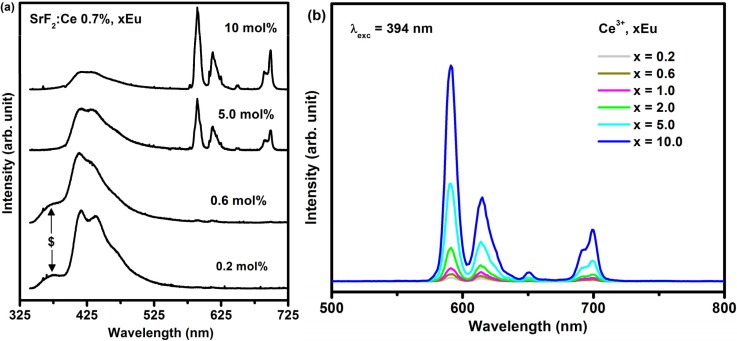
(**a**) PL spectra of SrF_2_:Ce^3+^ (0.7 mol%), xEu excited with the laser system with a 325 nm excitation wavelength and (**b**) 394 nm using the xenon lamp.

Figure 6a depicts the PL emission of Ce^3+^ (0.7%) co-doped SrF_2_:xEu (where *x* = 0.2%, 0.6%, 5% and 10%) excited with the He-Cd laser system with a 325 nm wavelength. The spectra also consisted of both the Eu^2+^ and Eu^3+^ emissions. A shoulder peak (marked with a dollar sign ($)) at a lower wavelength only appeared for the smaller dopant concentrations’ (0.2 and 0.6 mol%). This shoulder ($) is assigned to the 4f–5d emission of Ce^3+^, which is completely quenched at the higher Eu concentration. With an increasing concentration of the Eu ions the relative PL emission intensity of the Eu^2+^ gradually decreased and the Eu^3+^ emission intensity increased. The emission intensity of the Eu^3+^ has dramatically increased at the high Eu doping concentration. This can clearly be seen in Figure 6b where the Eu^3+^ emission intensity plotted as function of Eu concentration for the Eu co-doped Ce^3+^ system. It can be noticed that Ce^3+^ co-doped SrF_2_:Eu greatly enhanced the Eu^3+^ ions emission intensity at high Eu concentration. The increase of the Eu^3+^ emission intensity with an increase in the Eu concentration can be attributed to an increase in the Eu^3+^/Eu^2+^ ratio in the presence of the Ce^3+^ ions. In the SrF_2_ crystal, the Sr^2^^+^ ion is located at the body centre of a cube of eight F^−^ ions. The trivalent Ln^3+^ ions normally replace the Sr^2+^ cation. The extra charge of the Ln^3+^ ions is compensated by F^−^ anion charges situated elsewhere in an interstitial site. With increasing Ln^3+^ concentration, some kind of structural deformation occurs, the Ln^3+^-F dipoles couple to dimers, trimers and higher aggregates. The interstitial F^-^ ions and vacancies on the normal F^−^ site compose cuboctahedral clusters [32]. However, at low Eu concentration (less than Ce^3+^ concentration), the clusters are not completely formed. Besides, compare with the size of the Eu^3+^ (0.107 nm), the size of the Eu^2+^ (0.125 nm) is much closer to the size of the Sr^2+^ (0.126 nm), and hence the reduction of Eu^3+^ to Eu^2+^ ions is favored because it could reduce the lattice distortion of the doped SrF_2_ crystal [33]. At high Eu concentration (bigger than the Ce^3+^ concentration), the dimensions of the Eu^3+^ ions cluster increased and hence the ratio of Eu^3+^/Eu^2+^ increased. The increase of the Eu^3+^ ions therefore increased the Eu^3+^ emission intensity.

**Figure 7:**
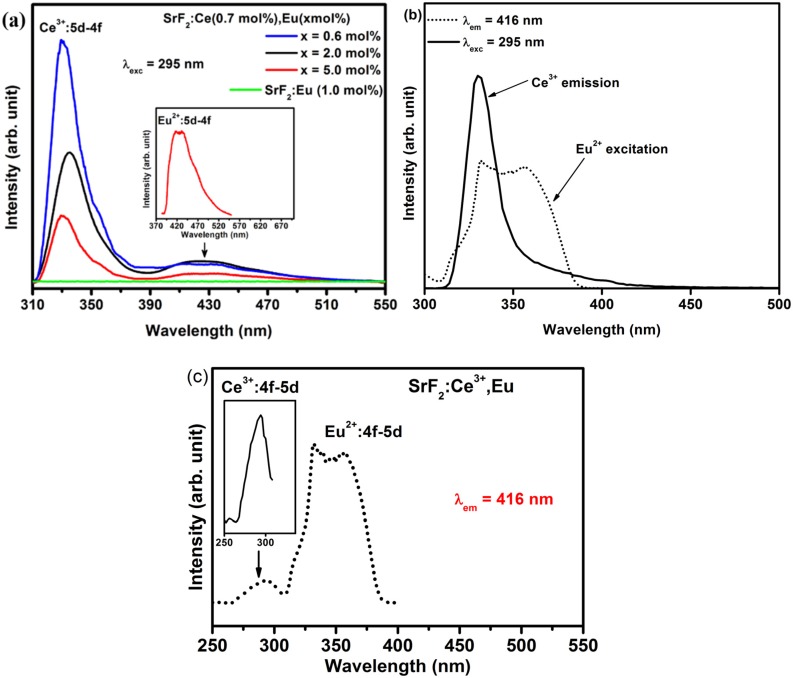
(**a**) PL emission spectra of Ce^3+^ and Eu^2+^ from SrF_2_:Ce^3+^ (0.7 mol%) with different Eu doping concentration as well as from Eu^2+^ in SrF_2_:Eu excited by an excitation wavelength of 295 nm; (**b**) Spectral overlap between Ce^3+^ emission and Eu^2+^ excitation and (**c**) excitation spectra of SrF_2_:Ce^3+^ (0.7 mol%), Eu (0.6 mol%) nanophosphors measured at an emission wavelength of 416 nm. The inset in (**a**) is the enlarge spectrum of the Eu^2+^emission ions and the inset in (**c**) is the enlarge Ce^3+^ excitation from SrF_2_:Ce^3+^ (0.7 mol%), Eu (5.0 mol%).

The PL emission spectra of the SrF_2_:Ce^3+^,Eu nanophosphor excited by the 295 nm excitation wavelength are plotted in Figure 7a. The broad emission band that is centered at a wavelength of 330 nm is a characteristic of the Ce^3+^ ion which is in agreement with the emission spectra for Ce^3+^ in Figure 4. The additional broad peak beside the Ce^3+^ emission that was centered at 416 nm is assigned to the Eu^2+^ ions in SrF_2_ (clearly shown in the inset graph of Figure 7a). The Eu^2+^ emission slightly increased before it decreased with increasing Eu concentration. In Figure 7a the emission spectrum of the SrF_2_:Eu without Ce excited at 295 nm is also shown. It clearly shows no Eu^2+^ emission has occurred. The presence of Eu^2+^ emission under 295 nm excitation, in the co-doped samples, is therefore evidence of an energy transfer process from Ce^3+^ to Eu^2+^. This process can occur in such material since the emission of Ce^3+^ overlaps the excitation spectra of Eu^2+^ (Figure 7b; SrF_2_:Ce^3+^ (0.7 mol%), Eu (0.6 mol%)). Such spectral overlap is a necessary condition for the occurrence of the energy transfer from Ce^3+^ to Eu^2+^. An efficient energy transfer from Ce^3+^ to Eu^2+^ in a fluoride crystal was previously demonstrated even for a very low concentration [34]. More evidence of energy transfer between Ce^3+^ and Eu^2+^ is shown in Figure 7c where the room temperature luminescence excitation spectra of SrF_2_:Ce^3+^ (0.7 mol%), Eu (0.6 mol%) nanophosphors are plotted. The excitation spectrum of Eu^2+^ (dotted line) not only consists of the Eu^2+^:4f^7^→4f^6^5d excitation transition but also the Ce^3+^ excitation band (clearly seen in the inset of the Figure 7c). All these results confirm the existence of energy transfer from Ce^3+^ to the Eu^2+^ ion.

**Figure 8:**
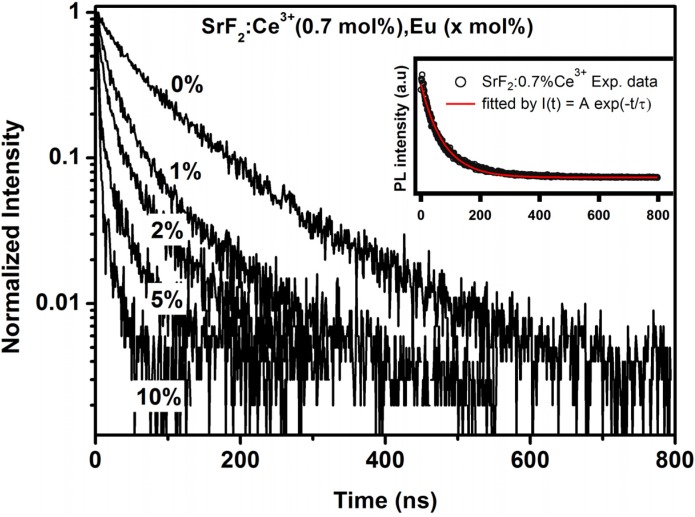
The decay lifetime of Ce^3+^ ions in the SrF_2_ host with an increase in Eu concentration. The inset graph shows the decay curve of 0.7% Ce^3+^ in SrF_2_ fitted to a single-exponential fitting function.

Results obtained from the luminescence decay curves for Ce^3+^ emission also contributed further to the energy transfer process. The decay time of the donor ions does not change in the presence and absence of the acceptor ions if the radiative energy is dominant [35]. In the situation of non-radiative energy transfer the decay time of the donor ions gradually decreases with an increase in the acceptor concentration. The PL decay curves of Ce^3+^ with various Eu concentration are shown in Figure 8. The decay curve of the Ce^3+^ ions gradually decreased with an increase in the Eu concentration. The luminescence decay curve of Ce^3+^ singly doped SrF_2_ nanoparticles can well be fitted into a single-exponential function, shown in the inset of Figure 8, whereas the decay curve of the entire co-doped concentrations were fitted with a bi-exponential decay model [35,36]:

I(t) = A_1_ exp(−t/τ_1_) + A_2_ exp(−t/τ_2_) (1)

I(t) is the luminescence intensity at time t; A_1_ and A_2_ are constants; and τ_1_ and τ_2_ are the short- and long-decay components, respectively. The average lifetime constant (τ̽ ) can be calculated from the following equation:


τ̽ = (A_1_ τ_1_^2^ + A_2_ τ_2_^2^)/(A_1_ τ_1_ + A_2_ τ_2_) (2)

The lifetime of the Ce^3^^+^ doped SrF_2_ is determined to be 77.15 ns. This value is in good agreement with the reported value of Ce^3+^ in SrF_2_ [27]. In the Eu ions co-doped system, the average lifetime of the donor ion (Ce^3+^) decreased up to 8.2 ns at 10 mol% Eu concentration. This results confirm that the excitation energy of Ce^3+^ ions was transferred to the Eu^2+^ ions. The lifetime results for the Ce^3+^ ions in the SrF_2_ host strongly suggest that the energy transfer from Ce^3+^ to Eu^2+^ was non-radiative. The energy transfer efficiency from Ce^3+^ to Eu is defined by the following expression:

ɳ_ET_ = 1− τ/τ_0_ (3)

where τ and τ_0_ are the average lifetime of Ce^3+^ in the presence and absence of the Eu ions, respectively. The corresponding lifetime and energy transfer efficiencies are tabulated in Table 2. From Table 2, the energy transfer of Ce^3+^ increased gradually with an increase in the Eu concentration. The maximum energy transfer efficiency is about 89.4% for the sample doped with 0.7 mol% Ce^3+^ and 10 mol% Eu. An efficient energy transfer has occurred from Ce^3+^ to Eu^2+^. The emission of Eu^2+^ has slightly increased before it decreased with increasing Eu concentration due to the decrease of the Eu^2+^ ratio in the SrF_2_ host. In our previous investigation of SrF_2_:Eu the Eu^2+^ ion was, however, found to be unstable when irradiated by a YAG laser. The Eu^2+^ ion’s PL emission intensity rapidly decreased with time and this result made the SrF_2_:Eu nanophosphor an unsuitable candidate for several applications, such as white light-emitting diodes and wavelength conversion films for silicon photovoltaic cells [10]. 

**Table 2:** Lifetime of the 5d–4f transition of Ce^3+^ (330 nm) and the Ce^3+^-Eu energy transfer efficiency (ɳ_ET_) in SrF_2_ matrix.

Eu concentration (mol%)	τ (ns)	ɳ_ET_ (%)
0	77.15	0
1	46.3	40
2	31.9	58.6
5	16.05	79.2
10	8.2	89.4

## 3. Experimental Section

Doped and un-doped SrF_2_ phosphor samples were synthesised by the hydrothermal method. For the hydrothermal process, all chemical reagents were of analytical grade and were used without further purification. For a typical synthesis, 1 mmol of Sr(NO_3_)_2_ was first dissolved in 30 mL distilled water, followed by 5 mmol of C_10_H_14_N_2_O_8_.2H_2_O (Na_2_EDTA, ethylenediamine tetraacetic acid disodium salt) and 2 mmol of NaBF_4_ under constant stirring. After further magnetic stirring for 10 min the solution was transferred into a 125 mL autoclave lined with Teflon, heated at 160 °C for one hour and naturally cooled down to room temperature [37]. The product was collected by centrifugal and washed with water and ethanol. Finally, the product was dried for 10 h in an oven at 60 °C. Ce^3+^ and Eu co-doped SrF_2_ samples were prepared by the same hydrothermal technique. Eu(NO_3_)_3_(H_2_O)_5_ and Ce(NO_3_)_3_(H_2_O)_6_ were used as sources for the Eu and Ce dopants, respectively.

The phosphors were characterized by X-ray diffraction (XRD) (Bruker AXS Gmbh, Karlsruhe, Germany) (Bruker Advance D8 diffractometer with Cu K_α_ radiation (λ = 0.154 nm)) to identify the crystalline structure of the powder. Auger spectra were collected with a PHI 700 Scanning Auger Nanoprobe (ULVAC-PHI Inc, Chanhassan, MN, USA) equipped with a scanning Auger microscope (SAM). The field emission electron gun used for the SAM analyses was set at: 2.34 A filament current; 4.35 kV extractor voltage and 381.4 µA extractor current. With these settings a 25 kV, 10 nA electron beam was obtained for the Auger analyses. The electron beam diameter was about 10 nm. An IonTof time of flight secondary ion mass spectrometer (TOF-SIMS) instrument (ION-TOF Gmbh, Muenster, Germany) equipped with a Bi primary ion source was used to characterize the nanophosphor materials for their chemical composition and dopants distribution. In spectroscopy mode, the system equipped with a DC current of 30 nA and a pulsed current of 1 pA at 30 kV with a heating current of 2.95 A and emission current of 0.8 μA was used. High resolution X-ray photoelectron spectroscopy (XPS) was obtained with a PHI 5000 Versaprobe system (ULVAC-PHI Inc, Chanhassan, MN, USA). A low energy Ar^+^ ion gun and low energy neutralizer electron gun were used to minimize charging on the surface. A 100 μm diameter monochromatic Al Kα X-ray beam (*hν* = 1486.6 eV) generated by a 25 W, 15 kV electron beam was used to analyze the different binding energy peaks. The pass energy was set to 11 eV giving an analyzer resolution ≤0.5 eV. Multipack version 8.2 software (ULVAC-PHI Inc, Chanhassan, MN, USA) was utilized to analyze the spectra to identify the chemical compounds and their electronic states using Gaussian-Lorentz fits. Photoluminescence spectra (PL) were collected using a Cary Eclipse fluorescence spectrophotometer (Varian Ltd, Mulgrave Victoria, Australia) equipped with a xenon lamp and also with a He-Cd laser PL system with a 325 nm excitation wavelength. Luminescence decay curves were recorded by using a NanoLED with a 335 nm excitation wavelength and repetition rate of 1 MHz. All measurements were performed at room temperature.

## 4. Conclusions

As-prepared SrF_2_:Eu,Ce nanophosphors were successfully synthesised with the hydrothermal technique. The average crystallite size that was calculated by using Scherrer’s equation was found to be 7.6 nm for the host sample. Dopant ions were intended to decrease the particle size of the host. The Auger spectra confirmed the presence of Sr, F, Eu and Ce elements in the host matrix. Photoluminescence properties of Ce^3+^ and Eu co-doped SrF_2_ nano-phosphor have been investigated. A possible efficient energy transfer from Ce^3+^ to Eu^2+^ ions was demonstrated. From the PL decay curves the energy transfer efficiency was calculated to be 89.4% for the SrF_2_: 0.7 mol% Ce^3+^, 10 mol% Eu sample.
